# Laparoscopic transabdominal preperitoneal repair of inguinal bladder hernia: a case report

**DOI:** 10.11604/pamj.2024.48.31.43628

**Published:** 2024-05-30

**Authors:** Ghassane El Omri, Omar Lazrak, Hamza Rais, Abdeljalil Heddat

**Affiliations:** 1 Department of Urology, Cheikh Khalifa International University Hospital, Mohammed VI University of Sciences and Health (UM6SS), Casablanca, Morocco

**Keywords:** Inguinal bladder hernia, bladder hernia, inguinal hernia, laparoscopic transabdominal preperitoneal approach, case report

## Abstract

Inguinal bladder hernia is a rare clinical condition, and only a small number of reported cases have been treated by laparoscopic surgery. We report a case of a patient aged 65-year-old who presented to our outpatient care unit for a right inguinal swelling. Computer tomography (CT) imaging showed a direct inguinal hernia with bladder and epiploic content. We performed a laparoscopic transabdominal preperitoneal repair, which involved carefully reducing the bladder's protrusion from the hernial orifice. Subsequently, a mesh prosthesis was employed to treat the right inguinal hernia. This case represents an unusual instance of a successful laparoscopic repair for a right direct inguinal bladder hernia.

## Introduction

Inguinal bladder hernias (IBH´s), a relatively uncommon clinical condition, constitute a small fraction, ranging from 0.5% to 4% of all inguinal hernias, with a notable predilection for those over 50 years of age [1]. The classification system distinguishes them into three categories: intraperitoneal, paraperitoneal and extraperitoneal [2]. Many cases of open surgery have been reported, but there are fewer documented cases and a lack of in-depth studies on the suitability of laparoscopic surgery for inguinal hernias of the bladder. We report a case of right direct inguinal bladder hernia in a 65-year-old patient who was successfully treated using a laparoscopic transabdominal preperitoneal (TAPP) approach for repair.

## Patient and observation

**Patient information:** a 65-year-old male presented in the outpatient care unit for right inguinal swelling. The patient had a history of high blood pressure treated with angiotensin-converting enzyme inhibitors.

**Clinical findings:** during the clinical examination, the patient was hemodynamically and respiratory stable, remaining afebrile. The abdomen was found to be soft and non-distended, featuring a 5 cm right-sided direct inguinal hernia, which displayed firm consistency and reducibility. The hernia was non-pulsatile and showed an increase in protrusion upon coughing, without any signs of skin inflammation or concurrent vomiting. Digital rectal examination revealed a mildly benign prostatic hyperplasia (BPH), estimated at 30 grams, and showed no evidence of suspicious nodules.

**Timeline:** the symptoms began one month before admission, with the progressive appearance of a right inguinal swelling that gradually increased in size. Subsequently, the patient started to present irritative low urinary tract symptoms (LUTS) including pollakiuria and urgency. The exacerbation of urinary symptoms concomitant with hernia's progression prompted the patient to seek consultation at the outpatient care unit.

**Figure 1:**
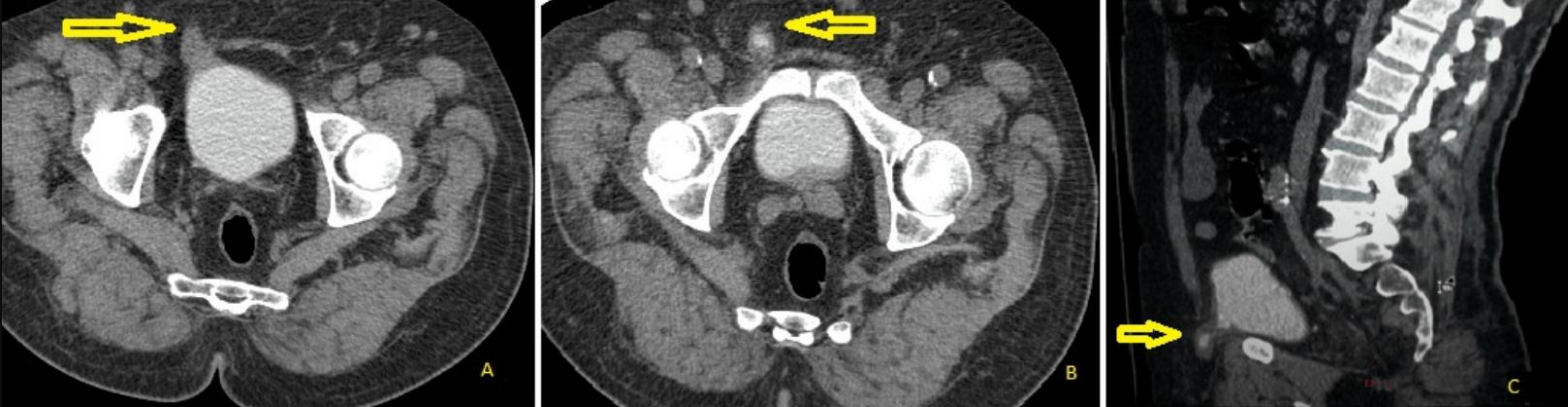
A-C) CT scan showing right direct inguinal hernia containing a bladder horn in axial and sagittal views

**Diagnostic assessment:** the abdomino-pelvic computer tomography scan revealed a direct inguinal hernia with bladder and epiploic content (Figure 1).

**Therapeutic intervention:** the patient underwent surgery one week following the consultation. The procedure involved repairing the inguinal hernia using a laparoscopic trans-abdomino-pre-peritoneal approach (TAPP), with the application of a polypropylene mesh. The operation was performed under general anesthetic with antibiotic prophylaxis.

**Figure 2:**
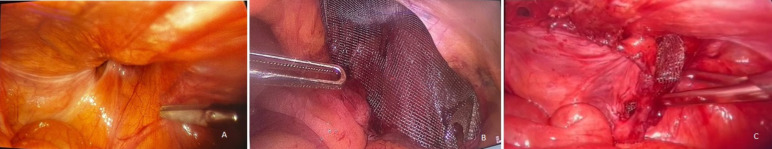
A) laparoscopic exploration revealing the presence of a large right direct inguinal hernia containing a bladder horn; B) placement of polypropylene mesh; C) covered mesh by the parietal peritoneum tissue

The procedure started with the placement of the first 12 mm trocar at the para-umbilical location. Following pneumoperitoneum insufflation, two 5 mm trocars were positioned under visual control in the iliac fossae, adhering to triangulation principles. During the identification of various elements of the spermatic cord, laparoscopic exploration revealed the presence of a large right direct inguinal hernia containing a bladder horn (Figure 2A). Subsequently, a wide anterior parietal peritoneal opening was performed, extending from the anterosuperior iliac spine to the tent of the homolateral umbilical artery. Careful dissection of the hernia sac was carried out, followed by verification of bladder integrity through bladder filling with 0.9% saline solution. The second phase involved the placement of a 15x15 cm polypropylene mesh (Figure 2B). The mesh was covered by the parietal peritoneum tissue and secured using a 5 mm Protack clip (Figure 2C).

**Follow-up and outcomes:** the patient had an uneventful post-operative recovery. The bladder catheter was removed the following day, and the patient was discharged two days after the operation. The patient had a follow-up consultation 15 days after the operation, during which it was observed that the incision was clean, the dressing was dry, and there were no signs of parietal infection. A positive clinical progress was noted, and LUTS disappeared.

**Patient perspective:** initially, I was quite concerned about the inguinal swelling, particularly the symptoms related to the lower urinary tract. However, thanks to the surgery, my condition has significantly improved. I would like to express my gratitude to the surgical team who provided excellent care for my condition.

**Patient consent:** an informed consent for the publication of this article in a journal was obtained from the patient.

## Discussion

Initially described in 1951 by Levine [3], IBH of inguinal hernias, accounting for only 1-4% of reported cases. Its incidence rises to 10% in obese patients over the age of 50 [1]. The hernial sac may contain either a diverticulum, a segment of the bladder, or the entire bladder [4]. The hernia is most often direct [5], occurring on the right side, with a 70% predominance in males [6]. Bladder hernias are classified into three types: intraperitoneal, where the bladder is entirely contained within the peritoneal sac; Paraperitoneal, where the herniated bladder is enveloped by a peritoneal sac on a singular side, lacking complete containment within it; And extraperitoneal, where no peritoneum covers the bladder [6]. The most common type is the paraperitoneal, while the least common is the extraperitoneal [6]. In our case, the patient had a right direct paraperitoneal bladder hernia, which is the most frequently observed type. This frequency is consistent with the findings of Hasegawa's retrospective study [7] involving 1126 cases of inguinal hernias. Among the 32 cases with a bladder hernia (2.8%), 12 were intraperitoneal (37.5%), 15 were paraperitoneal (46.9%), and only 5 were extraperitoneal (15.6%). The mechanism of inguinal bladder herniation involves the pulling of the bladder and the formation of a peritoneal sac through a weak point in the abdominal fascia [8]. Several factors contribute to the pathophysiological processes of bladder hernias, including advanced age, obesity, weakening of the pelvic musculature, decreased bladder tone, and bladder outlet obstruction caused by conditions such as benign prostatic hyperplasia or urethral stricture [8].

The diagnosis of bladder hernia can be challenging, given that the majority of patients are asymptomatic [4]. In symptomatic patients, in addition to the classic signs of a simple inguinal hernia, certain non-specific indicators may suggest an associated bladder hernia. These signs include irritative LUTS (pollakiuria, urinary urgency, nocturia), obstructive symptoms (dysuria, weak stream), and hematuria [2]. While these symptoms can also be attributed to other urinary pathologies such as BPH or a urinary tract infection, they are frequently encountered in instances of bladder hernias [2]. A study conducted by Branchu *et al*. [9]. involved a systematic review of more than 64 patients, revealing that among the 76% of patients with symptoms (n = 49), 48% presented with lower urinary tract symptoms. Additionally, the presence of Mery's sign is a significant diagnostic indicator for a bladder hernia [4]. This sign involves a two-stage micturition process facilitated by pressure on the hernial arch, with the hernia disappearing after emptying the bladder [9]. Mery's sign was not observed in our patient; however, the presence of LUTS was noted.

While most cases of inguinal hernia do not typically require routine preoperative radiological imaging, specific diagnostic examinations can provide valuable diagnostic and preoperative information. Ultrasound serves as the initial and readily accessible diagnostic modality, though it may have limitations in detecting small bladder hernias, especially the extraperitoneal subtype, which carries a higher risk of bladder injury [7]. For cases with a strong suspicion of inguinal bladder hernia, voiding cystourethrography is recommended, as it can reveal characteristic 'dumbbell' or 'dog-ear' shapes of the bladder [8]. CT is indicated for obese men aged 50 years or older with inguinal swelling and bladder lithiasis [4]. It proves especially beneficial in assessing the dimensions, placement, and contents within the hernia sac. and for identifying any associated pathologies or complications, such as hydronephrosis or strangulation [2]. Cystoscopy is essential for assessing the lower urinary tract and ureteral orifices and should be considered in cases of macroscopic hematuria to rule out an additional bladder tumor [4].

Several complications can arise from an inguinal bladder hernia, including urinary tract infections, obstructive uropathy (vesico-ureteric reflux and hydronephrosis), acute kidney injury, bladder stones, and even bladder strangulation, which can lead to bladder ischemia and infarction, which may necessitate subtotal cystectomy [6]. Furthermore, the primary complication associated with inguinal bladder hernia repair is bladder injury, which can result in persistent post-operative urine leakage, urine secretion from the surgical wound, hematuria, and the formation of fistulas [2]. This underscores the significance of preoperative diagnosis in preventing both intraoperative and postoperative complications. A study conducted by Khan *et al*. [2]. reported that the majority (77%) of bladder hernias were diagnosed intraoperatively, with only 7% diagnosed preoperatively and 16% diagnosed postoperatively due to complications.

Surgical repair of the hernia following bladder reduction currently represents the standard treatment. This procedure entails intraoperative reduction or, less commonly, resection of the bladder, followed by herniorrhaphy [8]. Currently, bladder resection is recommended only in cases involving bladder wall necrosis, a genuine herniated bladder diverticulum, a constricted hernia neck, or the existence of a tumor within the herniated bladder [6]. The selection of the surgical approach is contingent upon the surgeon's preference, the patient's local status, and overall health [4]. Open surgical repair with mesh usage fulfills all therapeutic objectives, including hernia removal, prevention of recurrences, reduction of postoperative pain, and the provision of postoperative comfort for rapid recovery, leading to favorable postoperative results. Nevertheless, laparoscopic repair offers specific advantages in cases of bladder hernia repair by reducing the risk of bladder injury [5]. Surgeons can confirm the presence of a hernial sac at the outset of laparoscopy in the TAPP procedure, eliminating the possibility of misidentifying the protruded bladder as a hernial sac [10]. Additionally, the existence of bladder injury can be readily verified by injecting saline or indigo carmine through a urinary catheter [10]. However, it is important to note that laparoscopic TAPP hernia repair has its limitations. In instances where a substantial portion of the bladder protrudes, the reduction of the protruded bladder may present challenges. In such instances, considerations should be made for the placement of an additional port to facilitate the repositioning of the protruded bladder or a potential conversion to open surgery [10].

## Conclusion

While bladder herniation is rare, it should be taken into consideration in elderly and obese patients who experience a sudden onset of lower urinary tract symptoms. Timely preoperative diagnosis plays a crucial role in preventing both intraoperative and postoperative complications. Laparoscopic TAPP hernia repair, holds promise as a standard option for treating inguinal vesical hernia while minimizing the risk of bladder injury.
